# LncRNA MALAT1 regulates METTL3-mediated PD-L1 expression and immune infiltrates in pancreatic cancer

**DOI:** 10.3389/fonc.2022.1004212

**Published:** 2022-09-21

**Authors:** Zhengwei Song, Xiaoguang Wang, Fei Chen, Qiuli Chen, Wenjun Liu, Xiaodan Yang, Xun Zhu, Xiaorong Liu, Peter Wang

**Affiliations:** ^1^ Department of Surgery, The Second Affiliated Hospital of Jiaxing University, Jiaxing, China; ^2^ Department of Research and Development, Zhejiang Zhongwei Medical Research Center, Hangzhou, China

**Keywords:** lncRNA, MALAT1, PD-L1, METTL3, TME, pancreatic cancer

## Abstract

Pancreatic cancer is the fourth leading cause of cancer death in the United States. The main methods of treating pancreatic cancer are surgery and chemotherapy, but the treatment efficacy is low with a poor prognosis. Immunotherapy represented by PD-1/PD-L1 has brought a milestone progress in the treatment of pancreatic cancer. However, the unique tumor microenvironment of pancreatic cancer presents challenges for immunotherapy. In addition, m6A is a common RNA modification and a potential molecular target in tumor therapy. The expression pattern of m6A in pancreatic cancer is still unclear. LncRNAs also play an essential role in pancreatic cancer development and treatment. In this study, we found that some m6A regulators were significantly elevated in pancreatic cancer and associated with the expression of PD-1/PD-L1. Moreover, we observed that METTL3 can increase the expression of PD-L1. Notably, METTL3 positively regulates the expression of lncRNA MALAT1 in pancreatic cancer cells. Strikingly, lncRNA MALAT1 increased the expression of PD-L1 in pancreatic cancer cells. This finding indicated that METTL3 regulated the expression of PD-L1 possibly *via* targeting lncRNA MALAT1 in pancreatic cancer cells. Lastly, MALAT1 governed the viability of pancreatic cancer cells. Taken together, lncRNA MALAT1 is involved in METTL3-mediated promotion of PD-L1 expression in pancreatic cancer.

## Introduction

Pancreatic ductal adenocarcinoma (PDAC) is one of the common malignant tumors of the pancreas and more than 90% of pancreatic cancer are exocrine PDAC (1). According to an epidemiology report, the five-year survival rate of pancreatic cancer after diagnosis is about 10%, and it is estimated that the pancreatic cancer will surpass breast cancer as the third leading cause of death (2). Cancer statistics suggest that there were 32,970 new cases of pancreatic cancer in men and 29,240 new cases of pancreatic cancer in women in the United States (3). The number of people who died from pancreatic cancer was 49,830 for men and 25,970 for women, respectively (3). Surgery and chemotherapy remain the mainstays of treatment for pancreatic adenocarcinoma (4). However, pancreatic cancer is aggressive with no obvious symptoms in the early stage. Most PDAC patients are late to be treated when diagnosed, and less than 20% of the patients are eligible for surgery (5). For unresectable patients, chemotherapy is the main treatment, but its efficacy is not ideal, and the median OS (Overall survival) is basically less than 1 year (6). Recently, immunotherapy, represented by immune checkpoint inhibitors (ICIs), has brought milestone progress to tumor treatment. However, ICIs are almost completely wiped out in pancreatic cancer, and most of them have failed in phase I and II clinical trials (7).

However, studies have shown that pancreatic cancer has a special tumor microenvironment (TME), which brings challenges to immunotherapy and deserves further study (8, 9). Tumor immune microenvironment (TIME) refers to the internal environment in which tumor cells are generated and live, which includes not only tumor cells themselves but also fibroblasts, immune and inflammatory cells, and glue cells that are closely related to tumor cells. However, compared with other cancers, pancreatic cancer has a unique TIME, which presents challenges for immunotherapy, and this may be one of the reasons why programmed death (PD-1)/PD ligand 1 (PD-L1) therapy is not highly sensitive to pancreatic cancer. PD-1, a 288 amino acid type 1 transmembrane protein, is often expressed on the surfaces of several immune cell types, while PD-L1, a 290 amino acid type 1 transmembrane protein, is expressed on hematopoietic cells (10, 11). Pancreatic tumor cells promote the activation of peripheral stromal cells and immunosuppressive cells, including regulatory T cells (Tregs), bone-marrow derived inhibitory cells (MDSCs), and tumor-associated macrophages (TAMs). At the same time, they secrete a series of cytokines and chemokines that cause these cells to flock to the tumor site. On the other hand, activated stromal cells generate a large amount of extracellular matrix that forms a fibrous “barrier” around pancreatic tumor cells, preventing effector cells (T and NK cells) from infiltrating into the tumor which allows tumor cells to evade immune surveillance. Activated immunosuppressive cells secrete immunosuppressive factors and express ligands (e.g., PD-L1 and B7-1/2), forming an immunosuppressive microenvironment. This plays an important role in the occurrence, development, invasion, metastasis and drug resistance of pancreatic cancer (12). The antitumor immune response is a complex, multistep process (13). Therefore, the mechanism of TIME should be further studied to explore new and potentially beneficial targets to improve the efficacy of pancreatic cancer immunotherapy.

Meanwhile, m6A is the most common RNA modification in eukaryotic RNA and plays an important role in cancer progression (14). It has been demonstrated that m6A modification is a dynamic and reversible process, which is composed of methyltransferase complex (Writers), demethylase (Erasers) and function managers (Readers) (15). It is believed that the function of m6A writers is stability of mRNA (2). A recent study show that there are 28 m6A regulators, including METTL3, METTL14, METTL16, WTAP, RBM15, RBM15B, ZC3H13, VIRMA, CBLL1, ZCCHC4, LRPPRC, ELAVL1, YTHDC1, YTHDC2, YTHDF1, YTHDF2, YTHDF3, HNRNPC, HNRNPA2B1, EIF3A, EIF3H, IGF2BP1, IGF2BP2, IGF2BP3, CBLL1, PRRC2A, FTO, ALKBH5 (16). m6A modification plays a role in pre-mRNA splicing, 3’-end processing, nuclear output, translation regulation, mRNA decay and miRNA processing, and its dynamic reversible changes control and determine cell growth and differentiation, suggesting that abnormalities of m6A and modified proteins may also produce pathological effects in the occurrence and progression of tumors (17). Besides, as the most common modification in mRNA, m6A links epigenomics with tumorigenesis and development, and affects the processes of tumor stem cell self-renewal and differentiation, proliferation and apoptosis, invasion and metastasis, drug resistance, and immunosuppression. Therefore, m6A is involved in m6A-modified key proteins that are expected to be potential molecular targets for cancer diagnosis and treatment and drug development. For instance, a study examining DNA and RNA methylation status in circulating tumor cells (CTCs) from lung cancer patients demonstrated for the first time elevated m6A modification levels in CTCs from lung cancer patients (18).

LncRNA, one type of noncoding RNA, participates in tumorigenesis and progression (19–22). LncRNA MALAT1 has been reported to regulate pancreatic oncogenesis. The expression of MALAT1 was highly elevated in PDAC compared with the adjacent normal specimens (23). MALAT1 expression was linked to invasion, tumor stage, poor survival, tumor size and metastasis in PDAC patients (23, 24). Moreover, MALAT1 enhanced invasion, migration and viability of pancreatic cancer cells *via* reduction of EMT and cancer stem cells as well as induction of apoptosis and cell cycle arrest (25). The role of MALAT1 in PDAC development is not fully elucidated. The expression pattern and pathophysiological role of m6A in pancreatic cancer remain unknown. In addition, the association between m6A methylation modulator and PD-L1 remains unexplored. Therefore, the main purpose of this paper aims to analyze the relationship between m6A RNA methylation regulators, PD-L1 and MALAT1 in pancreatic cancer.

## Materials and methods

### Data collection and m6A-related regulators

The data was collected from The Cancer Genome Atlas (TCGA) database and the basic information including age, gender, grade, stage, T, M and N of 178 samples was obtained. In this study, we collected 24 m6A-related regulators, including writers, erasers and readers. The writers include RBM27, METTL3, WTAP, RBM15, ZC3H7B, CAPRIN1 and METTL14. The erasers include FTO and ALKBH5. The readers include YTHDF1, YTHDF2, YTHDF3, IGF2BP1/2/3, YTHDC1/2, IGF2BPs, KIAA1429 (VIRMA), EIF3A, EIF3H, HNRNPC, HNRNPA2B1, LRPPRC, ELAVL1 and PRRC2A (26). To determine the interaction of these 20 regulators, they were searched in the Genes/Protein database to gain a preliminary understanding of their biological functions (27).

### Bioinformatic analysis

Consensus clustering is a method of providing quantitative evidence for determining the number and members of possible clusters in a data set, such as microarray gene expression (28). This approach is widely used in cancer genomics. In this study, we adopted this method to explore two PDAC clusters and their association with clinicopathological parameters. Gene set enrichment analysis (GSEA) 3.0 was used to predict the underlying downstream pathways of the two clusters (29). Immune score, stromal score, and tumor purity of each sample were calculated by using the ESTIMATE algorithm (30).

Genes with significant (p<0.01) prognosis were screened from DEGs using univariate COX regression analysis, and gene prognosis models were established by lasso-cox regression. The K-M curve was drawn using the R package survival, and the survival difference between different groups was calculated by the log-rank test to draw the K-M curve. The ROC of the model was calculated using the R package time ROC. Finally, the independent prognostic ability of risk scores was tested by univariate and multivariate Cox regression models. Groups will be divided into two groups including high-risk group and low-risk group by evaluating the distribution of clinical case characteristics using the R package “heatmap”. This study adopted Cox regression models to assess whether the risk score combining with other clinical characteristics, could be an independent prognostic factor.

### Cell culture and transfection

The human pancreatic cancer cell lines, BxPC-3 (with epithelial properties) and PANC-1 (with more mesenchymal properties), were cultured in DMEM medium supplemented with and 10% FBS and 1% penicillin/streptomycin solution. The cells were cultured in an incubator with 5% CO_2_ at 37°C. Human MALAT1 cDNAs were subcloned into pcDNA3 vector. Human METTL3 cDNAs were subcloned into pLenti-C-mGFP vector. Specific small hairpin RNAs (shRNAs) targeting METTL3 (shMETTL3) or MALAT1 (shMALAT1) and the control shRNA (shNC) were obtained (GenePharma, Shanghai, China).

### Quantitative real-time reverse transcription-PCR

Total RNA was extracted from pancreatic cancer cells using 1ml TRLzol Reagent. Then, RNA was used for reverse transcription and PCR was performed using SYBR Green Kit Data were analyzed by the ΔΔCt approach. GAPDH was used as the control. MALAT1: FW: GGA TCC TAG ACC AGC ATG CC; RV: AAA GGT TAC CAT AAG TAA GTT CCA GAA AA (31). The detailed method for PCR was described previously (32).

### Western blotting analysis

The pancreatic cells were lysed by RIPA buffer and a bicinchoninic acid (BCA) assay was used for detection of protein qualification. After proteins were separated onto SDS-PAGE, the proteins were transferred onto a PVDF membrane. The membrane was incubated with 5% non-fat milk and then incubated with METTL3 (1:1000), PD-L1 (1:1,000) or Tubulin (1:1,000) antibody for overnight at 4°C. The membranes were washed by TBST and then incubated with the secondary antibody for 1 h. Then, ECL method was used to examine the protein expression (33).

### Cell viability assay

The treated pancreatic cancer cells were cultured in 96-well plates for 48 and 72 hours. The viability of pancreatic cancer cells was determined by CCK8 assay as described previously (34). Briefly, 10 μL CCK8 reagent was added to each well and incubated for 2.5-3 hours in a cell culture incubator. The OD450 values were obtained by the microplate reader.

### Statistical analysis

In this study, the Pearson correlation coefficient was widely used to measure the degree of correlation between two variables, and its value was between -1 and 1. To assess the impact of M6A-related risk characteristics on the prognosis of PDAC, we compared the prognostic differences between the high-risk and low-risk groups. Kaplan-Meier plotter was used to analyze the relationship between gene expression profile and survival information in PATIENTS with PDAC. In addition, multivariate Cox regression analysis was performed to determine prognostic factors for PDAC patients. Student t test was used to validate significance between two groups. ANOVE was used to validate significance among three or more groups. P <0.05 is considered statistically significant.

## Results

### Expression of m6A RNA Methylation Regulators in PDAC

**Figure 1:**
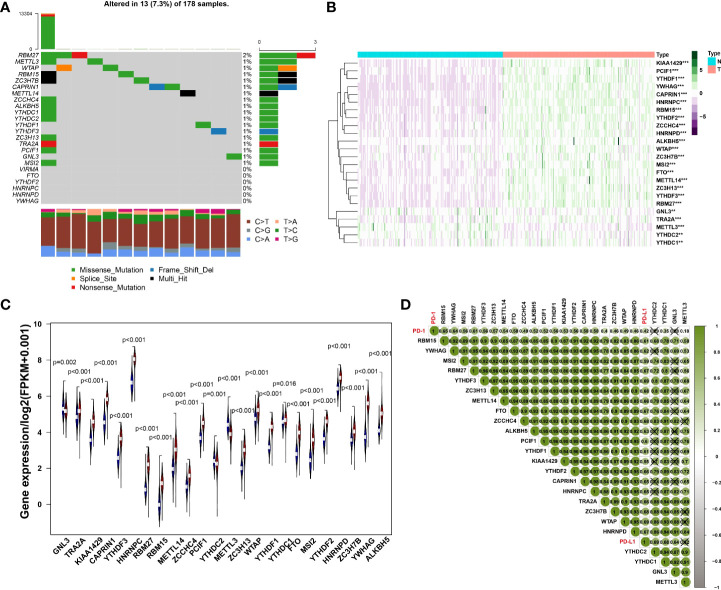
**(A)** The mutation frequency in each regulator. **(B)** Heatmap of m6A RNA methylation regulator expression level in each sample. **p<0.01; ***p<0.001. **(C)** The expression difference of m6A RNA methylation regulator between tumor and normal samples. **(D)** Correlation among PD-1, PD-L1 and m6A RNA methylation regulators.

Initially, we analyzed the frequency of mutations in 24 expressed m6A regulators and found that 18 regulators all had a mutation frequency of 1%~2%. Mutations occurred in 7.3% of the 178 samples, the most common mutation being missense mutation (**Figure 1A**). To further understand the expression of m6A RNA methylation regulator in tumor and normal samples, the heatmap was conducted and the results have revealed that compared with normal samples, there were 24 regulators of m6A, whose expression was relatively higher in cancer tissues (**Figure 1B**). Besides, the expression difference of m6A RNA methylation regulators between tumor and normal samples was significant since the p-value is less than 0.05 (**Figure 1C**).

### Correlations among PD-L1, PD1, and m6A RNA methylation regulators

The results of Pearson correlation suggested that PD-1 is associated with 20 regulators, including RBM15, YWHAG, MSI2, RBM27, YTHDF3, ZC3H13, METTL14, FTO, ZCCHC4, ALKBH5, PCIF1, YTHDF1, KIAA1429, YTHDF2, CAPRIN1, HNRNPC, TRA2A, ZC3H7B, WTAP, HNRNPD, YTHDC1 and METTL3. The correlation coefficient of PD-1 and RBM15 was the strongest. Similarly, the correlation between PD-L1 and WTAP was the strongest with a correlation coefficient of 0.69, while the relationship between PD-L1 and KIAA1429 was the weakest with a correlation coefficient of 0.55. All regulators of PD-1, PD-L1 and M6A were positively correlated (**Figure 1D**).

### Analysis of consensus clustering

**Figure 2:**
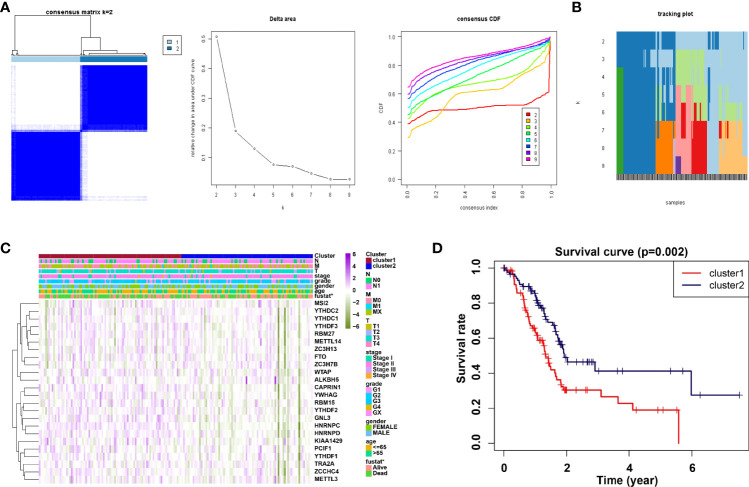
Correlation of consensus clustering for m6A RNA methylation regulators with the characteristics and survival of PDAC patients. **(A)** Consensus clustering matrix for k=2 (left panel); Consensus clustering cumulative distribution function (CDF) for k=2 to 9 (middle panel); relative change in area under CDF curve for k=2 to 9. **(B, C)** Heatmap of correlation of m6A RNA methylation regulators with characteristics of PDAC patients. **(D)** Kaplan-Meier curves of overall survival (OS) for patients.

Consensus clustering method was adopted to aggregates data such as transcriptome and proteome profiles. From **Figure 2A**, it is believed that k = 2 has the optimal clustering stability from k = 2 to 9. Then, the consensus clustering has identified the PDAC cohort of TCGA into two clusters and demonstrated their relationship with clinicopathological parameters (**Figure 2A**). Subsequently, the heatmap of correlation of m6A RNA methylation regulators with characteristics of PDAC patients and the tracking plot have been concisely displayed (**Figures 2B, C**). Furthermore, the overall survival (OS) for PDAC patients was analyzed by using Kaplan-Meier curves. It is noteworthy that cluster2 had a significantly higher survival rate than cluster1 (**Figure 2D**).

### Infiltrating levels of immune cell types in cluster1/2 with PDAC

**Figure 3:**
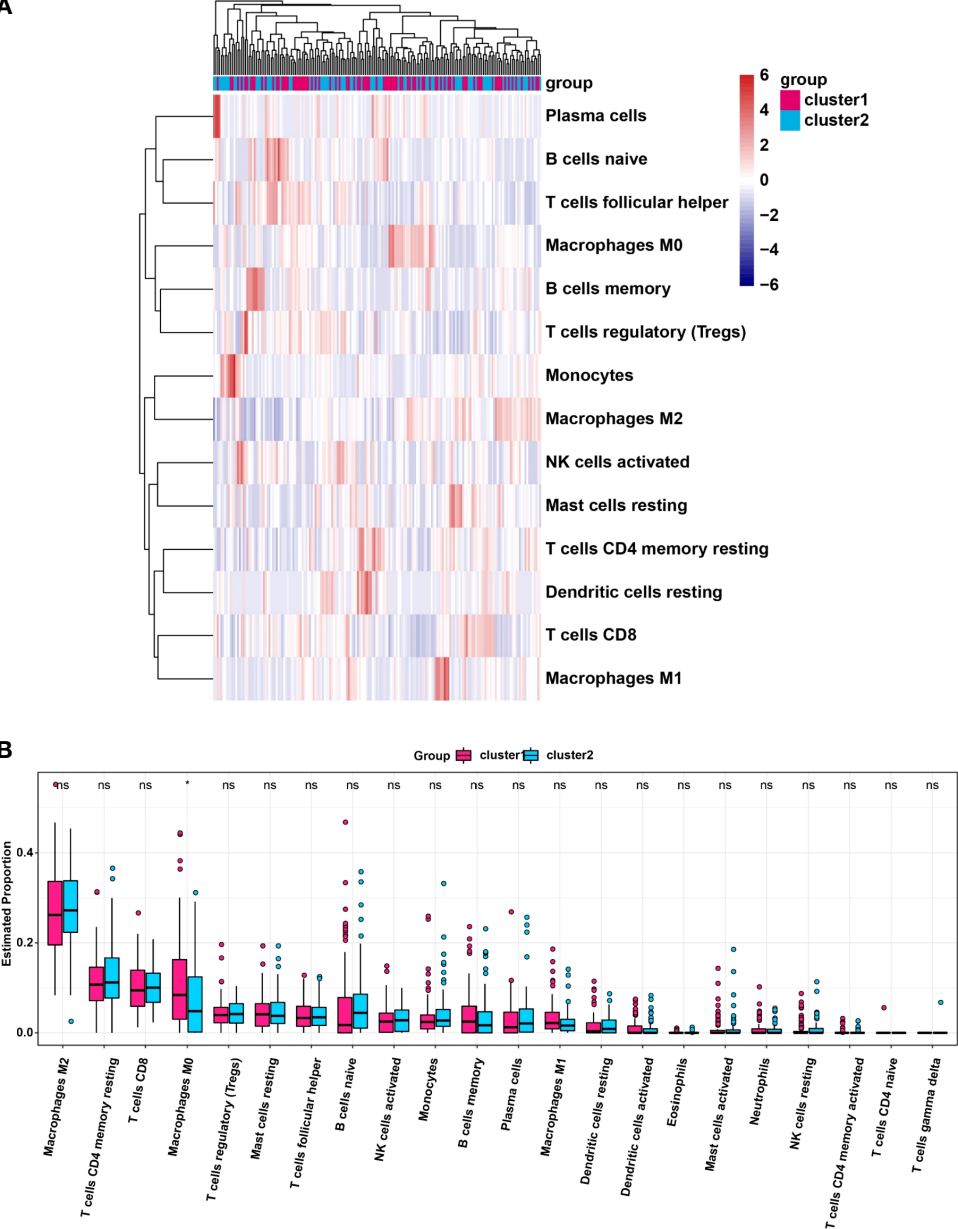
**(A)** Heatmap of infiltrating levels of various immune cells in cluster1/2 in pancreatic cancer. **(B)** Estimated proportion of 22 immune cell types in cluster1/2 in pancreatic cancer. *p<0.05; ns, no significance.

**Figure 4:**
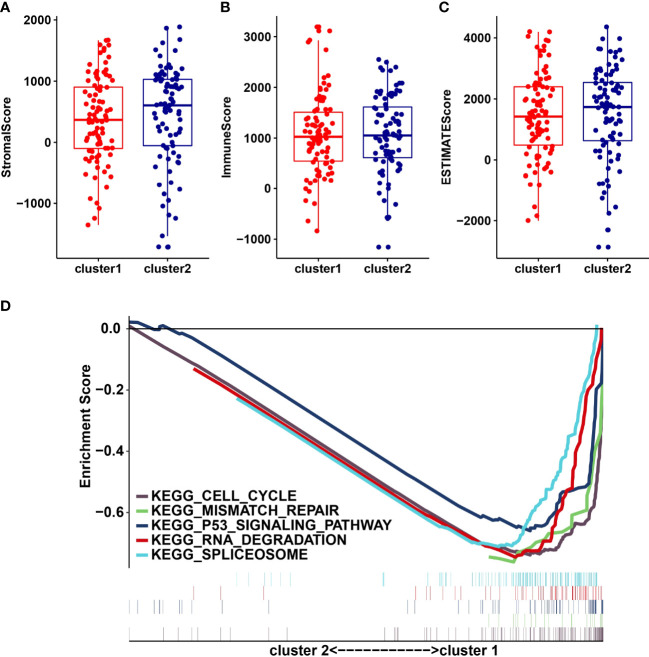
StromalScore **(A)**, ImmunoScore **(B)**, EstinateScore **(C)** in the cluster1/2 subtypes are illustrated. **(D)**: The signaling pathways are involved in cluster1 and cluster2.

We analyzed the infiltrating levels of various immune cells in cluster1/2 in PDAC and the results were displayed (**Figure 3A**). Meanwhile, the graph of estimated proportion of 22 immune cell types in cluster1/2 suggested that the estimated proportion of Macrophages MO of cluster1 was higher than that of Macrophages MO of cluster2, (**Figure 3B**). In order to further understand the infiltrating levels in cluster1/2 with PDAC, we compared the StromalScore, ImmuneScore and EstimateScore. We found the StromalScore and EstimateScore of cluster2 were higher than that of cluster1, which indicated that cluster2 had a higher degree of immune infiltration than cluster1 (**Figures 4A–C**). Moreover, cluster1 and cluster2 were involved in the following five signaling pathways: cell cycle, mismatch repair, p53 signaling pathway, RNA degradation and Spliceosome (**Figure 4D**).

### Construction and validation of prognostic characteristics of m6A regulators

**Figure 5:**
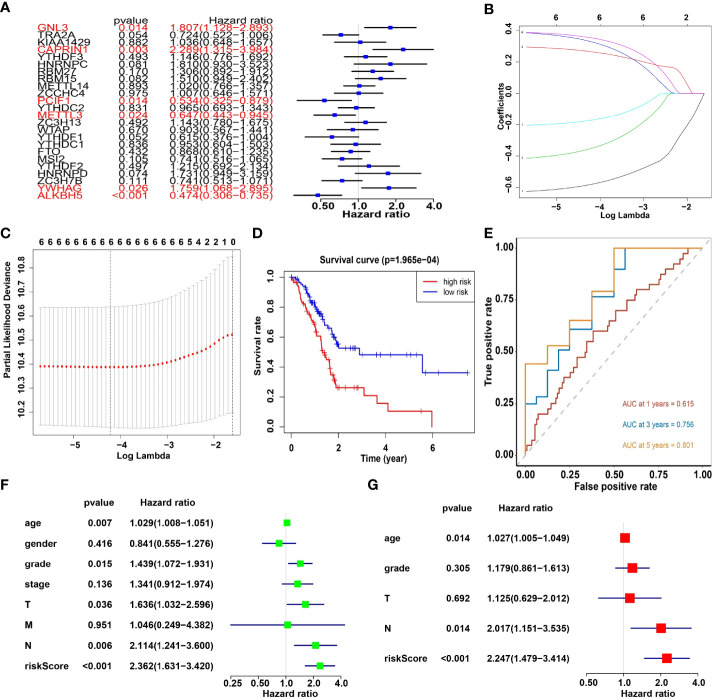
**(A)** Univariate analysis of 24 regulators. **(B, C)** LASSO Cox regression algorithm. **(D)** The Kaplan-Meier curve of high risk and low risk group. **(E)** Time-dependent ROC curves. **(F, G)** Univariate and multivariate Cox regression analysis of the risk scores in TCGA.

Univariate analysis of 24 m6A RNA methylation regulators was performed to identify genes, which may significantly associate with prognosis. Indeed, the results revealed that GNL3, CAPRIN1, PCIF1, METTL3, YWHAG and ALKBH5 were significantly associated with OS with hazard ratios of 1.807, 2.289, 0.534, 0.647, 1.759 and 0.474, respectively. The 95 percent confidence intervals were (1.128-2.893), (1.315-3.984), (0.325-0.897), (0.443-0.945), (1.068-2.895) and (0.306-0.735), respectively (**Figure 5A**). Overall, the hazard ratio of GNL3, CAPRIN1 and YWHAG was greater than 1, while the hazard ratio of PCIF1, METTL3 and ALKBH5 was less than 1. Then through the lasso regression algorithm, the coefficient of prognostic genes was identified (**Figures 5B, C**).

**Figure 6:**
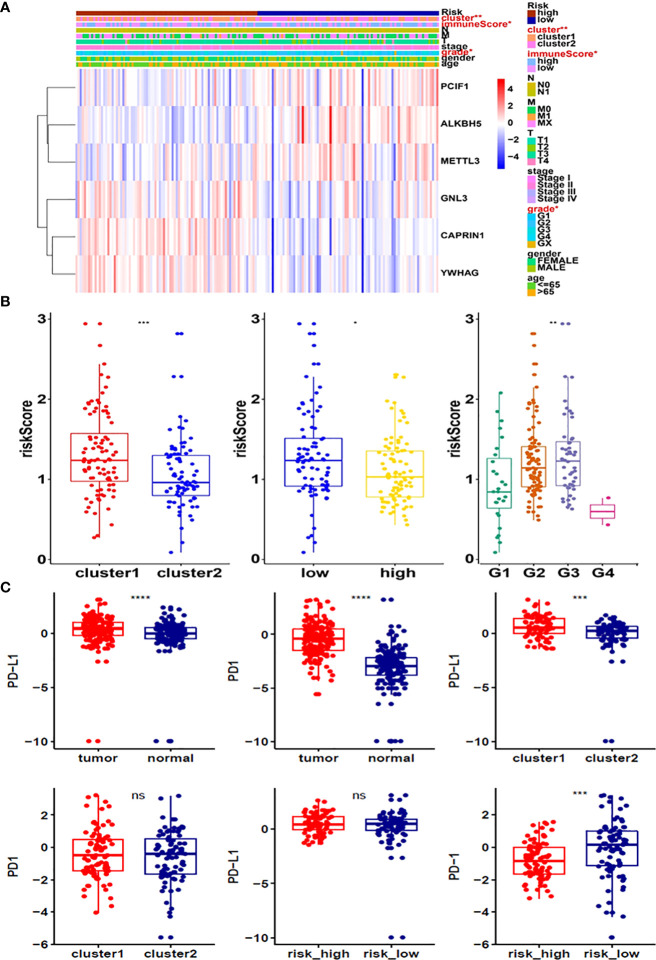
**(A)** Heatmap of clinicopathological features of pancreatic cancer cohort. **(B)** Distribution of risk scores stratified by cluster1/2. **(C)** The expression of PD-1 and PD-L1 in tumors, cluster1/2 and high/low-risk groups. *p<0.05; **p<0.01; ***p<0.001; ****p<0.0001. ns, no significance.

Moreover, survival analysis showed higher survival rates in the low-risk group than in the high-risk group (**Figure 5D**). The AUC at 1 years, 3 years and 5 years is 0.615, 0.756 and 0.801 (**Figure 5E**). Furthermore, to determine whether prognostic marker-based risk scores are independent prognostic indicators for pancreatic cancer patients, univariate and multivariate Cox regression analyses of risk scores were performed. The results proposed that N and risk score were independent prognostic indexes (p-value=0.014, HR=0.017; p-value<0.001, HR=2.247) (**Figures 5F, G**). The clinical features of PDAC cohort has been displayed in **Figure 6A**. The riskscore of cluster2 was higher than that of cluster1, and similarly, the riskscore of the low-risk group was higher than that of the high-risk group. Among G1, G2, G3 and G4 groups, G3 group had the highest riskscore, while G4 group had the lowest riskscore (**Figure 6B**). It is noteworthy that PD-1 and PD-L1 were highly expressed in pancreatic cancer cells compared with normal cells. Compared with cluster2, PD-L1 was highly expressed in cluster1(**Figure 6C**).

### METTL3 regulates the expression of PD-L1 and lncRNA MALAT1.

**Figure 7:**
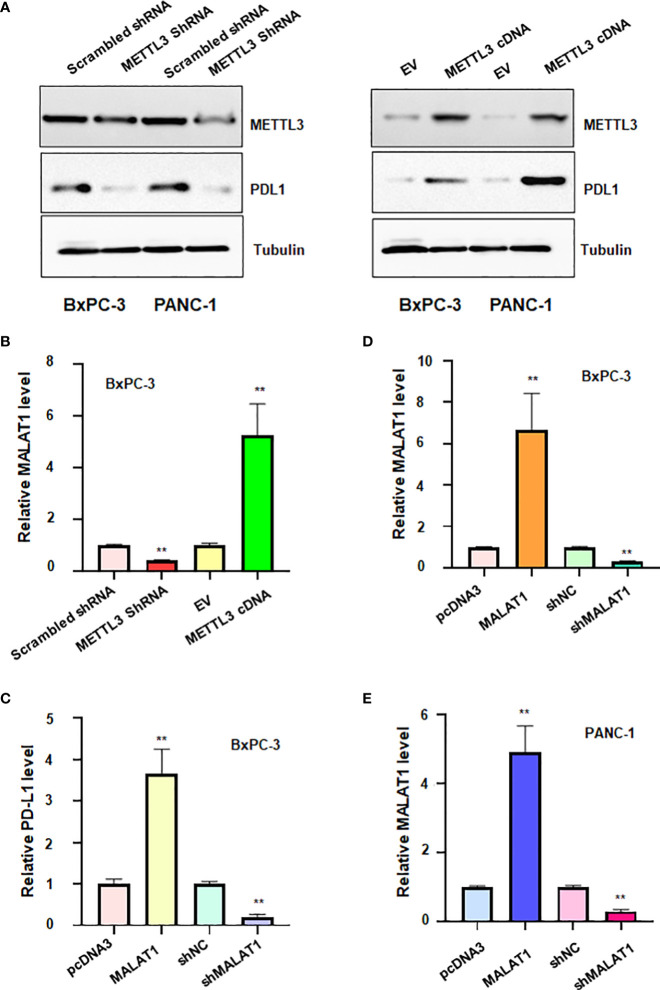
The relationship between METTL3, lncRNA MALAT1 and PD-L1 in PADC cells. **(A)** Western blotting was used to measure the expression of PD-L1 in BxPC-3 and PANC-1 cells after METTL3 modulation. **(B)** RT-PCR was used to measure the expression of lncRNA MALAT1 in BxPC-3 cells after METTL3 modulation. **(C)** RT-PCR was used to test the expression of PD-L1 in BxPC-3 cells after lncRNA MALAT1 changes. D-E: RT-PCR was used to measure the expression of MALAT1 in BxPC-3 **(D)** and PANC-1 cells **(E)** after MALAT1 modulation. **p<0.01.

To confirm the association between METTL3 and PD-L1 in pancreatic cancer cells, we transfected METTL3 cDNA and shMETTL3 plasmids to BxPC-3 and PANC-1 cells. We observed that overexpression of METTL3 increased the expression of PD-L1, whereas shMETTL3 infection led to downregulation of PD-L1 in pancreatic cancer cells (**Figure 7A**). It has been known that METTL3 can regulate the expression of lncRNA MALAT1. Next, we tested whether METTL3 modulation can govern the expression level of lncRNA MALAT1 in pancreatic cancer cells. Indeed, upregulation of METTL3 increased the MALAT1 level, and downregulation of METTL3 reduced the expression of MALAT1 in pancreatic cancer cells (**Figure 7B**). Moreover, MALAT1 overexpression increased PD-L1 expression level, while reduction of MALAT1 reduced PD-L1 level in pancreatic cancer cells (**Figure 7C**). Taken together, METTL3 regulates the expression of PD-L1 partly due to regulation of lncRNA MALAT1 in pancreatic cancer.

### LncRNA MALAT1 regulates viability of pancreatic cancer cells.

**Figure 8:**
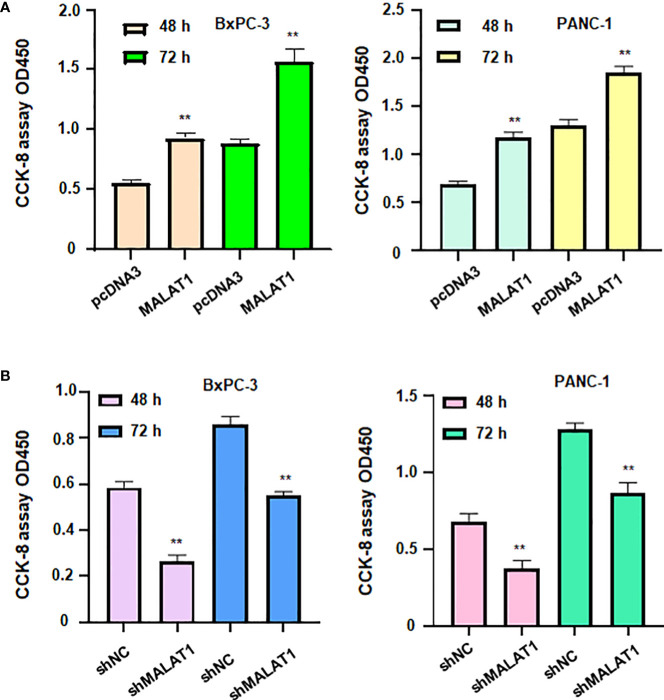
LncRNA MALAT1 regulates viability of pancreatic cancer cells. **(A)** CCK-8 assay was used to measure the viability of BxPC-3 and PANC-1 cells after MALAT1 overexpression. **(B)** CCK-8 assay was conducted to measure the viability of BxPC-3 and PANC-1 cells after MALAT1 downregulation. **p<0.01.

To define the role of lncRNA MALAT1 in regulation of viability of pancreatic cancer cells, we used shMALAT1 or MALAT1 cDNA to modulate the expression of MALAT1 in pancreatic cancer cells. We found that shMALAT1 transfection suppressed the expression of lncRNA MALAT1, while MALAT1 cDNA transfection elevated the expression of MALAT1 in pancreatic cancer cells (**Figures 7D, E**). Moreover, increased expression of MALAT1 promoted the viability of BxPC-3 and PANC-1 (**Figure 8A**). Furthermore, depletion of MALAT1 attenuated the cell viability at 48 h and 72 h in pancreatic cancer cells (**Figure 8B**). Altogether, lncRNA MALAT1 regulates viability of pancreatic cancer cells.

## Discussion

Pancreatic cancer is one of the common malignant tumors of the digestive tract and is known as the “king of cancer” in the field of tumor (35). The current treatment methods are mainly chemotherapy and surgery (36). However, the five-year survival rate after diagnosis of pancreatic cancer is about 10%, and it is one of the malignant tumors with poor prognosis (37). Immunotherapy of PD-1/PD-L1 has brought the hope for the treatment of pancreatic cancer (38). However, studies have shown that pancreatic cancer has a special tumor immune microenvironment, which brings challenges to immunotherapy and deserves further study (8). m6A methylation is the most common form of mRNA modification and involves in tumorigenesis (39). However, the role of m6A methylation in pancreatic cancer and the relationship between m6A, PD-1 and the infiltration of TIME in pancreatic cancer were unelucidated.

This study analyzed the relationship between m6A RNA methylation regulators, PD-L1, prognosis and TIME in pancreatic cancer. We found a total of 24 m6A genes that were highly expressed in the tumor samples. PD-1/PD-L1 was significantly associated with 20 m6A regulators. Subsequently, we used consensus clustering to identify two subgroups (cluster 1 and cluster 2) and found that patients in cluster2 displayed better prognosis than cluster1. Furthermore, cluster1 and cluster2 may be associated with cell cycle, p53 pathway, mismatch repair, RN degradation and Spliceosome. Notably, we identified risk signatures, including GNL3, CAPRIN1, METTL3, YWHAG, ALKBH5 and PCIF1.

A recent study found that there is a significantly increasing of METTL3 expression in PDAC cells (40), and this is consistent with our study. Our findings suggested that the expression difference of m6A RNA methylation regulator between tumor and normal samples was significant. Moreover, other evidence suggested that the upregulation of METTL3 can promote proliferation and invasion of pancreatic cancer (41). The chemical resistance in tumor cells will increase because of the rising expression of METTL3 (42). More specifically, a study pointed out that hypomethylation of the METTL3 promoter leads to overexpression of METTL3, which cooperates with NF-κB activating protein (NKAP) to coordinate m6A modification of the primary transcript of miR-25, making it mature miR-25-3p. Moreover, miR-25-3p inhibits PHLPP2 and activates oncogenic AKT-p70S6K signaling, and promotes the occurrence and progression of pancreatic cancer (43). METTL3 expression plays an important role in the TIME of pancreatic cancer (44, 45).

METTL3 has been reported to regulate the expression of PD-L1 in various cancer types. One study showed that METTL3 increased the expression of PD-L1 and intensified the malignant phenotype in oral squamous cell carcinoma (46). Another group identified that METTL3 can upregulate the expression of PD-L1 mRNA in breast cancer cells (47). In line with this report, METTL3 also elevated the PD-L1 mRNA in bladder cancer cells (48). In the present study, we found that METTL3 had a weak association with PD-L1 expression in pancreatic cancer patients. Moreover, METTL3 positively regulates the expression of PD-L1 in pancreatic cancer cells. METTL3 has been known to upregulate the expression of MALAT1 in several cancer types. METTL3 promoted the stability of MALAT1 and enhanced the glioma progression (49). METTL3 targeted MALAT1/miR-26b/HMGA2 axis and caused EMT and promotion of migration and invasion in breast cancer (50). METTL3 regulated MALAT1/E2F1/AGR2 pathway and subsequently controlled Adriamycin resistance in breast cancer (51). We also found that METTL3 controlled the expression of MALAT1 in pancreatic cancer cells.

LncRNA MALAT1 upregulated the expression of PD-L1 *via* sponging miR-195, leading to promotion of tumorigenesis in diffuse large B cell lymphoma (52). MALAT1 elevated the PD-L1 expression level *via* inhibition of miR-200a-3p, resulting in non-small lung cancer progression (53). Our data showed that MALAT1 can regulate the expression of PD-L1 in pancreatic cancer cells. LncRNA MALAT1 has been found to maintain the cancer stem cell-like properties in pancreatic cancer cells, including self-renewing ability, chemoresistance and angiogenesis (54). Han et al. reported that MALAT1 interacted with EZH2 and suppressed E-cadherin expression, leading to EZH2-induced invasion and migration in pancreatic cancer (55). Li et al. found that MALAT1 facilitated tumor cell metastasis and proliferation *via* the promotion of autophagy in pancreatic cancer (56). Zhang et al. revealed that miR-216a triggered apoptosis and G2/M arrest in pancreatic cancer cells *via* inhibition of MALAT1 expression (57). MALAT1 downregulation retarded pancreatic cancer progression *via* targeting Hippo-YAP signaling pathway (58). Moreover, MALAT1 was involved in efficacy of gemcitabine treatment in pancreatic cancer patients (59). Recently, MALAT1 was identified to govern pancreatic cancer progression *via* modulation of miR-129-5p (60). In the current study, we found that depletion of MALAT1 reduced cell viability, whereas overexpression of MALAT1 enhanced viability of pancreatic cancer cells.

## Conclusion

In summary, this study is the first time to apply a bioinformatic approach to describe the relationship between m6A and PD-L1 and the TIME in pancreatic cancer. There is a limitation that this work mainly used a bioinformatic strategy to explore the association among m6A, PD-L1 and TME in pancreatic cancer. The role of METTL3 in pancreatic cancer development should be validated in animal study and clinical tissue samples. The mechanism of lncRNA MALAT1-mediated pancreatic oncogenesis should be dissected. How METTL3 regulates PD-L1 expression *via* regulation of lncRNA MALAT1 is required to be determined in the future.

## Data availability statement

The original contributions presented in the study are included in the article/Supplementary Material. Further inquiries can be directed to the corresponding authors.

## Author contributions

ZS, XW, FC, WL, QC, XY and XZ performed the experiments, analyzed the data. ZS, QC and XL wrote the manuscript. PW edited the manuscript and supervised this study. All authors read and approved the final manuscript.

## Funding

This research was supported by Natural Science Foundation of Zhejiang Province (No.LY21H160046).

## Conflict of interest

The authors declare that the research was conducted in the absence of any commercial or financial relationships that could be construed as a potential conflict of interest.

## Publisher’s note

All claims expressed in this article are solely those of the authors and do not necessarily represent those of their affiliated organizations, or those of the publisher, the editors and the reviewers. Any product that may be evaluated in this article, or claim that may be made by its manufacturer, is not guaranteed or endorsed by the publisher.
